# Genetic Testing and Analysis in Breast Cancer Patients in Greece

**DOI:** 10.7759/cureus.72897

**Published:** 2024-11-02

**Authors:** Ioannis Rellias, Drakoulis Yannoukakos, Florentia Fostira, Paraskevi Apostolou, Andreas Pampanos, Dimitrios Loutradis, George Daskalakis, Constantine Dimitrakakis

**Affiliations:** 1 First Department of Obstetrics and Gynecology, National and Kapodistrian University of Athens, Alexandra Hospital, Athens, GRC; 2 Breast Surgery, Euroclinic Athens, Athens, GRC; 3 Human Molecular Genetics Laboratory INRASTES (Nuclear &amp; Radiological Sciences and Technology, Energy &amp; Safety), National Centre for Scientific Research Demokritos, Athens, GRC; 4 Genetics, Alexandra General Hospital, Athens, GRC

**Keywords:** brca1, brca2, breast cancer, brip1, chek2, family history, genetics, germline pathogenic variants, msh2, tp53

## Abstract

Introduction

Genetic testing for inherited breast cancer predisposing pathogenic variants (PVs) inform treatment choices and guide clinical management strategies in breast cancer patients.

Methods

The study enrolled 146 patients, sourced from the Breast Unit database of the First Department of Obstetrics and Gynecology at the University of Athens, Alexandra Hospital. Blood samples were collected for genetic testing, utilizing a detailed 94-cancer gene panel. The results were then descriptively correlated with the clinicopathological data of the patients.

Results

In this study, 17 PVs were identified across several genes, *BRCA1* (10), *BRCA2* (3), *CHEK2* (3) and *TP53* (1), while 4 variants of unknown clinical significance (VUSs) were found in *BRCA2* (1), *CHEK2* (1), *BRIP1* (1), *MSH2* (1). This corresponds to a prevalence rate of 11.6% (17/146). Cascade testing was conducted for 7 of the 17 positive families, resulting in 11 individuals being tested, of whom 3 tested positive. ﻿

Conclusions

Our study findings on the selected Greek population align with current literature. Genetic testing following National Comprehensive Cancer Network (NCCN) guidelines offers valuable insights for patients and their families. This information enhances counseling and identifying germline PVs could refine treatment strategies, potentially improving prognostic outcomes.

## Introduction

Breast cancer is the most diagnosed cancer in women [1,2]. In 2022, breast cancer was the leading cancer site among women in Europe, with 530,000 cases and it remains the primary cause of cancer-related deaths among women worldwide [1,3]. Germline pathogenic variants (PVs) in *ATM*, *BRCA1*, *BRCA2*, *CHEK2*, and *PALB2* are detected in 5-7% of unselected women with breast cancer in the general population and are associated with a significantly increased risk of breast cancer in unaffected women [4,5]. This is consisted with the fact that in approximately 20-30% of patients with a personal or family history of the disease, a PV is identified. In a recent study involving 306,147 women, around 11% reported having a first-degree family history of breast cancer, with 3,885 cases of breast cancer diagnosed [6]. Including a family or personal history of other cancers (such as ovarian, prostate, or pancreatic) can raise the percentage of breast cancer cases with positive family history to as much as 20-30% [7].

The American Society of Clinical Oncology (ASCO) recommends offering *BRCA1/2 *genetic testing to all patients newly diagnosed with breast cancer who are 65 years or younger. For patients older than 65, testing should be considered based on factors such as personal or family history, ancestry, or eligibility for poly (ADP-ribose) polymerase (PARP) inhibitor therapy [8]. Additional guidelines for genetic testing of other high or low penetrance genes are provided by ASCO [8]. Furthermore, the American Society of Breast Surgeons has recommended since 2019 that genetic testing be offered to all breast cancer patients. This testing should include *BRCA1*, *BRCA2*, and *PALB2* genes, along with other relevant genes based on the patient's clinical situation and family history [9].

In that context the genetic testing of breast cancer patients and the genetic counseling has become a necessity in clinical practice. In the current study we are presenting genetic testing and analysis results of breast cancer patients in a specialized gynecological hospital.

## Materials and methods

The study included 146 adult female patients from two departments affiliated with the National and Kapodistrian University of Athens: the Breast Unit of the First Department of Obstetrics and Gynecology and the Department of Clinical Therapeutics, both located at Alexandra Hospital in Athens, Greece. All the participants had a confirmed diagnosis of breast cancer (either invasive or in situ carcinomas), as recorded in pathology reports from the hospital’s pathology laboratory archives. The study received approval from the hospital's institutional review board.

The study involved presenting the research details to eligible patients, who were then given the choice to participate based on voluntary informed consent. Each participant received a copy of the consent form to review. Prior to signing, ample time was provided for discussion and to address any questions they had. Patients opting to join were enrolled in the study. Diagnosis of breast cancer was determined using standard clinical, radiological, and histological criteria [10]. For each eligible patient, a thorough medical history was compiled, which included demographic and clinicopathological data such as ethnicity, age at diagnosis, tumor characteristics (including stage, size, and grade), lymph node involvement, hormone receptor status, and human epidermal growth factor receptor type 2 (HER2) status. Additionally, detailed family cancer histories were gathered in collaboration with the Genetics Department at Alexandra Hospital. A 10ml blood sample was collected from each participant for germline genetic testing. Each sample was assigned a unique identification number (patient number) by the laboratory.

Genetic testing was conducted following the procedures outlined in previous studies, with a summary of the steps provided below [11-15].

Screening for Greek BRCA1 founder variants

Initial genetic testing focused on the detection of five *BRCA1* founder mutations specific to the Greek population, as well as one recurrent pathogenic *BRCA1* variant, as previously reported [4,11]. This included three large genomic rearrangements (LGRs) affecting the *BRCA1* C-terminus domain, all exclusive to individuals of Greek descent, along with three single nucleotide variants: *c.5212G>A*,* c.5266dupC*, and *c.5251C>T* [11-15].

Genomic capture and massively parallel sequencing using TruSight Cancer Panel

Germline DNA underwent enzymatic fragmentation, followed by adaptor tagging, indexing, and targeted capture of 1,736 regions across 94 cancer predisposition genes using the TruSight Cancer Panel, in accordance with the manufacturer’s instructions (Illumina, USA). The panel includes the following genes: *AIP, ALK, APC, ATM, BAP1, BLM, BMPR1A, BRCA1, BRCA2, BRIP1, BUB1B, CDC73, CDH1, CDK4, CDKN1C, CDKN2A, CEBPA, CEP57, CHEK2, CYLD, DDB2, DICER1, DIS3L2, EGFR, EPCAM, ERCC2, ERCC3, ERCC4, ERCC5, EXT1, EXT2, EZH2, FANCA, FANCB, FANCC, FANCD2, FANCE, FANCF, FANCG, FANCI, FANCL, FANCM, FH, FLCN, GATA2, GPC3, HNF1A, HRAS, KIT, MAX, MEN1, MET, MLH1, MSH2, MSH6, MUTYH, NBN, NF1, NF2, NSD1, PALB2, PHOX2B, PMS1, PMS2, PRF1, PRKAR1A, PTCH1, PTEN, RAD51C, RAD51D, RB1, RECQL4, RET, RHBDF2, RUNX1, SBDS, SDHAF2, SDHB, SDHC, SDHD, SLX4, SMAD4, SMARCB1, STK11, SUFU, TMEM127, TP53, TSC1, TSC2, VHL, WRN, WT1, XPA *and* XPC*. Amplified libraries were assessed both qualitatively and quantitatively using the Fragment Analyzer (Advanced Analytical Technologies, Germany). Sequencing was performed on a MiSeq platform using the Standard V2 kit with 150 base paired-end reads. FASTQ, binary alignment map (BAM), and variant call format (VCF) files were generated through the Illumina MiSeq Reporter, and variant annotation was conducted using the human reference genome GRCh38 with VariantStudio V.3 (Illumina). The minimum base coverage was 50×, with amplicon coverage at 100×, and a mean read depth of 182×. All PVs were validated by Sanger sequencing [11-15].

Multiplex Ligation-Dependent Probe Amplification

Due to the limitations of next-generation sequencing (NGS)-based testing in detecting large deletions and duplications outside the *BRCA1* founder LGRs in the Greek population, the SALSA Multiplex Ligation-Dependent Probe Amplification (MLPA) kits P002, P045, P190, and P056 were utilized to screen for LGRs in the *BRCA1*, *BRCA2*, *CHEK2*, and *TP53* genes, respectively, following the manufacturer's instructions [11-15].

Variants were interpreted and classified according to the guidelines established by the American College of Medical Genetics and Genomics (ACMG) [16].

## Results

Blood samples were collected from 146 patients regardless of age, family history, or the presence of invasive or pre-invasive disease. The average age at diagnosis was 44 years. The majority of participants were of Greek origin (137 out of 146, 93.8%), with the remaining patients representing diverse ancestries: two Romanian, two Russian, one Albanian, one Ukrainian, one Ethiopian, one Moldavian, and one Armenian.

The study included 138 cases of invasive disease and 8 cases of pure ductal carcinoma in situ (DCIS). 12 cases presented with bilateral invasive disease, while two showed invasive disease in one breast and DCIS in the other. Among patients with bilateral disease, all but three exhibited the same expression of hormonal receptors (HRs) and HER2 in both breasts. In the remaining three cases, luminal B disease was observed in one breast and triple-negative disease in the other.

The distribution of breast cancer histological types among 146 cases was as follows: invasive ductal carcinoma (IDC) was the most prevalent, accounting for 111 cases (76%). Invasive lobular carcinoma (ILC) was the second most common type with 14 cases (9.6%). Other types included three cases of tubular carcinoma (2.1%), two cases of medullary carcinoma (including one bilateral case, totaling 1.4%), and seven cases classified as mixed types (4.8%). Among the mixed types, IDC combined with ILC in three cases, with metaplastic carcinoma in two cases, with mucinous carcinoma in one case, and with micropapillary carcinoma in one case. Additionally, there was one case of mucinous carcinoma, representing 0.7% of all cases.

The distribution of HR and HER2 expression among invasive breast cancer cases and DCIS is depicted in Table 1. For one case, immunohistochemistry (IHC) was not performed due to the small size of the preinvasive disease (DCIS 2 mm).

**Table 1:** Expression of ER, PR, HER2 proteins in invasive breast cancer cases and DCISs ER: Estrogen receptor, PR: Progesterone receptor, HER2: Human epidermal growth factor receptor type 2, DCIS: Ductal carcinoma in situ, N: number of cases as a fraction of the total

ER/PR/HER2 expression in invasive breast cancer cases	N	Percentage
ER(+) PR(+) HER2(-)	(67/138)	48.6%
ER(-) PR(-) HER2(-)	(30/138)	21.7%
ER(+) PR(+) HER2(+)	(20/138)	14.5%
ER(-) PR(-) HER2(+)	(9/138)	6.5%
ER(+) PR(-) HER2 (-)	(6/138)	4.3%
ER(-) PR(+) HER2(-)	(2/138)	1.4%
ER(-) PR(+) HER2(+)	(1/138)	0.7%
ER(+) PR(-) HER2(+)	(3/138)	2.2%
ER/PR expression in DCIS cases	N	Percentage
ER(+) PR(+)	(5/8)	62.5%
ER(-) PR (-)	(2/8)	25%
ER(+) PR (-)	(0/8)	0%
ER(-) PR(+)	(0/8)	0%

PVs were identified in 17 out of 146 patients, constituting 11.6% of the study population, consistent with existing literature, while 4 variants of unknown clinical significance (VUSs) were also characterized. Specifically, findings include 10 patients with PVs in *BRCA1*, 3 in *BRCA2*, 1 VUS in *BRCA2*, 1 pathogenic and 2 likely PVs in *CHEK2*, 1 VUS in *CHEK2*, 1 PV in *TP53*, and single instances of VUS in *MSH2* and *BRIP1*.

Out of the 17 families identified as positive, cascade testing was conducted in 7 families, involving 11 individuals, among whom 3 tested positive. Notably, the mother of patient 2469 carries a likely pathogenic *CHEK2* variant (*p.Gly167Arg*), the mother of patient 2548 is a carrier of a pathogenic *BRCA2* variant (*c.5110_5113delAGAA *(*exon 11*)), and the mother of patient 2740 is a carrier of a pathogenic *BRCA1* variant (*del exons 23-24*).

Among the 17 carriers, 8 (47%) were diagnosed with triple-negative breast cancer (TNBC), all before the age of 50. Among these cases, three had bilateral disease, and one had both breast and ovarian cancer. Most carriers also had a familial history involving breast, ovarian, pancreatic, or prostate cancer. Table 2 presents detailed clinical and pathological characteristics of individuals with positive genetic test results.

**Table 2:** Clinical-pathological characteristics of variant carriers Ca: Cancer; IDC: Invasive ductal carcinoma; R: right; L: left, ILC: Invasive lobular carcinoma; DCIS: Ductal carcinoma in situ; ER: Estrogen receptor; PR: Progesterone receptor; HER2: Human epidermal growth factor receptor type 2; LN: Lymph nodes; (-): negative; (+): positive; VUS: Variant of unknown clinical significance; low: Low hormone receptor expression In the LN column, the fraction represents the number of infiltrated nodes relative to the total number of nodes removed. * denotes that these cases were also reported by Zografos et al [2].

Patient	Diagnosis	Age of Diagnosis	Gene	Variant/Clinical Significance	Histology/Maximum Tumor Diameter	ER	PR	HER2	Grade	LN
2960	Ca breast	32 (R)	BRCA1	c.5266dupC/Pathogenic	IDC - R: 2.3 cm	R: (-)	R: (-)	R: (-)	R: III	R: 0/5
	Bilateral	35 (L)			IDC - L: 3 cm	L: (-)	L: (-)	L: (-)	L: ΙΙI	L: 0/9
2104	Ca breast bilateral, Ca ovary	49 (L) breast, 53 ovary, 59 (R) breast	BRCA1	p.Gly1738Arg (exon20)/Pathogenic	IDC - L: 1.3 cm	R: (-)	R:(-)	R: (-)	R: II	R:0/5
					IDC - R: 1.5 cm	L: (-)	L: (-)	L: (-)	L: ΙΙI	L:0/7
2469	Ca breast	35	CHEK2	p.Gly167Arg/Likely pathogenic	IDC: 0.5 cm	(+)	(+)	(-)	ΙΙΙ	0/3
1949	Ca breast	35	BRCA1	p.Gly1738Arg(exon20)/Pathogenic	IDC: 1 cm	(+)	(+)	(+)	ΙΙΙ	0/22
2215	Ca breast	34	BRCA2	c.1405_1406delGA (exon10)/Pathogenic	Mixed type (IDC/ILC): 4.3 cm	(-)	(-)	(-)	ΙΙΙ	0/4
2548	Ca breast	35	BRCA2	c.5110_5113delAGAA (exon11)/Pathogenic	IDC (& DCIS): 1.4 cm	(+)	(+)	(-)	ΙΙ	0/2
553*	Ca breast	32	BRCA1	c.5328delC/Pathogenic	IDC: 15.5 cm	(-)	(+)	(-)	ΙΙΙ	0/17
749*	Ca breast	33	BRCA1	c.3700_3704delGTAAA/Pathogenic	IDC: 4 cm	(-)	(-)	(-)	ΙΙΙ	0/46
964*	Ca breast	41 (L)	BRCA1	c.3700_3704delGTAAA/Pathogenic	Medullary - L: 1.8 cm	R: (-)	R: (-)	R: (-)	R: III	L: 1/21
	Bilateral	44 (R)			Medullary - R: 2.3 cm	L: (-)	L: (-)	L: (-)	L: ΙΙI	R:1/17
2754*	Ca breast	30	BRCA1	p.Arg1751X (exon 20)/Pathogenic	Medullary: 2.5 cm	(-)	(-)	(-)	ΙΙΙ	0/25
2740*	Ca breast	26	BRCA1	del exons 23-24/Pathogenic	IDC	(+)	(+)	(-)	ΙΙ-ΙΙΙ	7/12
2017	Ca breast	40	MSH2	p.Pro616Arg/VUS	IDC: 1.8 cm	(+)	(+)	(-)	Ι	0/4
2605	Ca breast	39	CHEK2	p.Gly167Arg/Likely pathogenic	IDC: 5 cm	(-)	(-)	(-)	III	1/53
750*	Ca breast	34	BRCA1	G1738R/Pathogenic	IDC: 5 cm (& DCIS)	(+) low (<10%)	(-)	(-)	III	8/13
1164	Ca breast	36	BRCA1	c.181T>G (p.Cys61Gly)/ Pathogenic	IDC: 3 cm	(-)	(-)	(-)	III	3/24
1927*	Ca breast	37	CHEK2	c.1100delC (p.Thr367MetfsX1)/Pathogenic	IDC: 5 cm	(+)	(+)	(-)	II	2/10
2122*	Ca breast	32	BRIP1	c.2285G > A (p.Arg762His)/VUS	IDC: 9 cm	(+)	(+)	(-)	III	22/34
3227*	Ca breast	37	BRCA2	c.8386C>T p.(Pro2796Ser)/VUS	IDC: 2 cm	(+)	(+)	(-)	II	0/10
1045*	Ca breast	37	CHEK2	c.1175C > T (p.Ala392Val)/VUS	IDC: 5 cm	(+)	(+)	(-)	III	4/6
9794181	Ca breast	33	TP53	c.473G>A/p.(Arg158His)/Pathogenic	IDC: 2.5cm	(+)	(+)	(-)	III	22/29
24000763	Ca breast	32	BRCA2	NM_000059:c.2808_2811del, p.(Ala938Profs*21)/Pathogenic	IDC: 4 cm	(+) 5% low	(-)	(-)	III	0/10

Table 3 presents detailed family history of individuals with positive genetic test results. 

**Table 3:** Family history of variant carriers Ca: cancer, y: years old, (m): maternal, (f): paternal, †: death, BC: Breast Cancer, PCa: Prostate Cancer CRC: Colorectal Cancer, No FH: negative family history for malignancies, VUS: Variant of Unknown Clinical Significance, (*) Denotes that these cases were also reported by Zografos E. et al [2].

BRCA1 germline variants	Clinical Significance	Patient number	Family History
c.181T>G - p.Cys61Gly (300T>G - C61G)	Pathogenic	1164	The mother diagnosed with BC at 35y. The mother’s sister diagnosed with BC at 40y.
c.3700_3704delGTAAA (3819del5) (p.Val1234Glnfs)	Pathogenic	749*, 964*	749: The mother diagnosed with BC at 31y. The mother’s sister diagnosed with endometrial Ca at 51y. 964: no FH.
c.5266dupC (5382insC)	Pathogenic	2960	The mother diagnosed with BC at 50y. The mother’s sister diagnosed with BC at 38y. The other mother’s sister diagnosed with Ca of unknown primary site at 45y. The maternal grandmother diagnosed with ovarian Ca and the paternal grandmother diagnosed with BC at 32y.
c.5212G>A- p.Gly1738Arg (5331G>A- G1738R)	Pathogenic	750*, 1949, 2104	750: The sister diagnosed with BC at 32y. The father’s sister diagnosed with ovarian Ca at 63y (†73y). The paternal grandmother diagnosed with BC at 70 (†75y). The mother’s sister diagnosed with abdominal Ca at 70y(†). 1949: The mother diagnosed with BCa at 50y. The maternal grandmother’s sister diagnosed with BC at 89y. 2104: The mother diagnosed with BC at 55y (†66y-new primary ca). The mother’s brother diagnosed with lung ca (†65y) and the maternal grandmother diagnosed with Ca of unknown primary site (†85y).
c.5251C>T - p.Arg1751X (5370C>T - R1751X)	Pathogenic	2754*	The mother diagnosed with BC at 53y. The paternal grandmother diagnosed with melanoma at 87y and the paternal grandfather diagnosed with PCa at 61y.
c.5328delC (5447delC) (p.Thr1777fs)	Pathogenic	553*	The maternal grandmother diagnosed with vaginal Ca at 90y. The father and the two paternal uncles diagnosed with PCa at 60y.
g.169527_ 180579del11052 (p.Gly1803_ Tyr1863del11052)	Pathogenic	2740*	The maternal grandfather diagnosed with lung Ca at 50y and the maternal grandmother diagnosed with endometrial Ca at 67y.
BRCA2 germline variants	Clinical Significance	Patient number	Family History
c.1405_1406delGA	Pathogenic	2215	The mother diagnosed with Ca of unknown primary site (†54y). The maternal grandmother diagnosed with CRC at 60y and the maternal grandmother’s brother diagnosed with liver Ca.
c.5110_5113delAGAA	Pathogenic	2548	The mother diagnosed with BC at 64y. The mother’s sister diagnosed with ovarian Ca at 64y. The mother’s 1st cousin from her father’s side diagnosed with BC at 42y.
c.2808_2811delACAA	Pathogenic	24000763	The paternal grandfather diagnosed with lung and PCa at 78 y.
c.8386C>T p.(Pro2796Ser)	VUS	3227*	No FH
CHEK2 germline variants	Clinical Significance	Patient number	Family History
c.1100delC (p.Thr367MetfsX1)	Pathogenic	1927*	No FH
c.499G>A (p.Gly167Arg)	Likely pathogenic	2469, 2605	2469: The father diagnosed with lymphoma at 67y. The paternal grandmother diagnosed with BC at 45y. The father’s brother diagnosed with CRC at 64y. Two grandmother’s brothers from the father's side diagnosed with Ca of unknown primary site. The paternal great-grandmother diagnosed with Ca of unknown primary site (†50y). Two paternal grandfather’s brothers diagnosed with lung Ca and 2 children of them had Ca of unknown primary site. Maternal grandmother diagnosed with liver Ca (†66y). 2605: No FH
c.1175C>T (p.Ala392Val)	VUS	1045*	No FH
TP53 germline variant	Clinical Significance	Patient number	Family History
c.473G>A / p.(Arg158His)	Pathogenic	9794181	The mother’s sister diagnosed with BC at 56 y.
MSH2 germline variant	Clinical Significance	Patient number	Family History
c.1847C>G (p.Pro616Arg)	VUS	2017	The maternal aunt diagnosed with BC at 55y. The mother diagnosed with non-Hodgkin's lymphoma at 55y. The maternal grandmother diagnosed with BC at 96y. The daughter of paternal grandmother’s sister diagnosed with BC at 60y.
BRIP1 germline variant	Clinical Significance	Patient number	Family History
c.2285G > A (p.Arg762His)	VUS	2122*	A 1st degree relative had PCa and a 2nd degree relative had BC.

**Figure 1:**
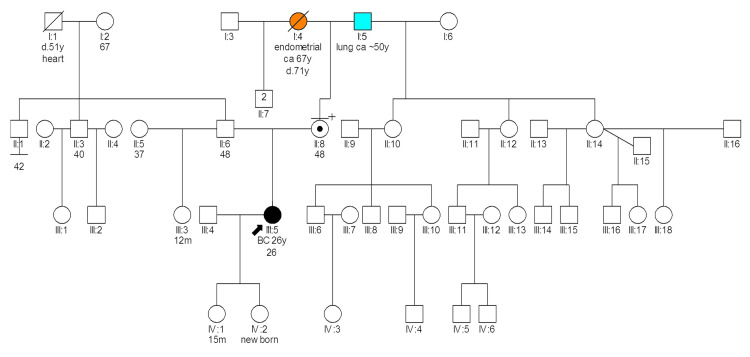
Pedigree of patient 2740 carrying the BRCA1 del exons 23-24 variant Latin numbering denotes the generation; d: death; y: age in years; Ca: Cancer; BC: Breast cancer; m: age in months

Patient 2740 (Figure 1) is a Greek individual with the *BRCA1* deletion of exons 23-24 variant. She was diagnosed with breast cancer at 26 years old, while 33 weeks pregnant. A modified radical mastectomy was performed, and pathology revealed three foci of IDC, the largest being 3 cm in size, graded II-III. Out of 12 excised axillary lymph nodes, 7 were positive for cancer (stage T2N2M0) [17]. IHC showed estrogen receptor (ER)/progesterone receptor (PR) positive and HER2 negative disease, with ki67 as 15%. The patient delivered via caesarean section at 35 weeks of gestation. Post delivery, she received adjuvant chemotherapy, radiotherapy, and is currently on hormonal therapy. Six years post treatment, she remains disease-free.

The patient’s family history shows limited malignancies. The patient’s mother, who also underwent cascade genetic testing, carries the same *BRCA1* PV. On the maternal side, the grandmother had endometrial cancer at 67, and the grandfather had lung cancer at 50. The other relatives from this side should be tested. 

**Figure 2:**
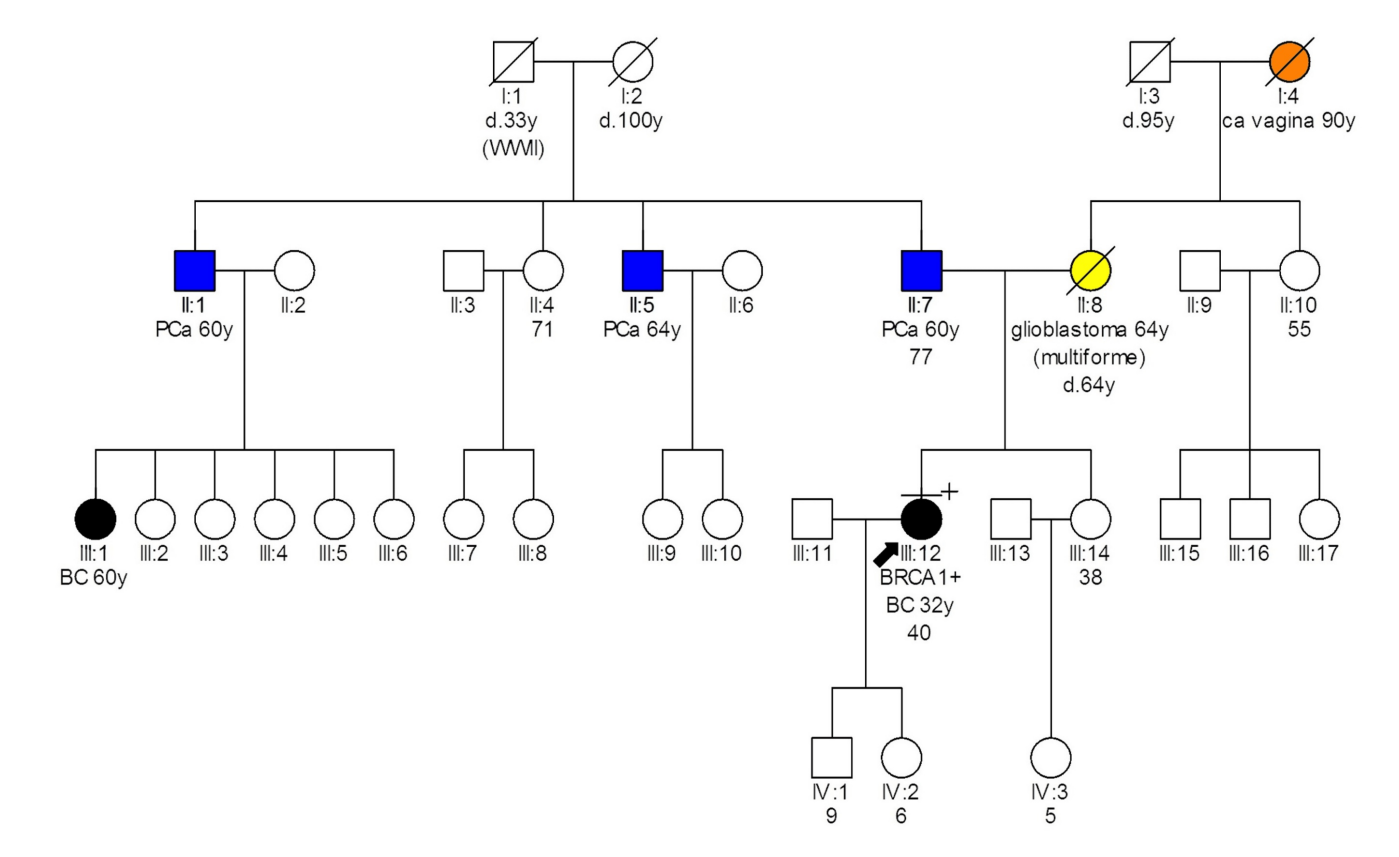
Pedigree of patient 553 carrying the BRCA1 c.5328delC variant Latin numbering denotes the generation; d: death; WWII: World War II; y: age in years; Ca: Cancer; Ca vagina: Vaginal cancer; PCa: Prostate cancer; BC: Breast cancer; BRCA1+: BRCA1 gene pathogenic mutation carrier

Patient 553 (Figure 2) carries the *BRCA1 c.5328delC *variant and was diagnosed with breast cancer at 32 years old, while she was 18 weeks pregnant. A modified radical mastectomy was performed during pregnancy. Pathology examination showed IDC grade III, size 15.5cm with 0 positive axillary lymph nodes out of 17 excised (stage T3N0M0) [17]. IHC examination revealed ER as 0%, PR as 3%, HER2 as 0, p53 as 90%, and ki67 as 90%. She underwent adjuvant chemotherapy during pregnancy and gave birth by caesarean section at 35 weeks of gestation. Thereafter she had adjuvant chemotherapy, radiotherapy, and hormonal therapy. She is currently free of disease 12 years after diagnosis. 

As we observe from the patient's pedigree (Figure 2), there are three prostate cancer cases in the family, the father and two paternal uncles, all at around 60 years of age. It is a malignancy that could be related to the specific variant that patient 553 carries. This patient has two children, each with a 50% likelihood of inheriting this genetic variant. Genetic counseling and testing for the offspring are recommended once they reach adulthood.

Patient 24000763, diagnosed with TNBC at the age of 32, underwent NGS using the 94-gene cancer panel as above mentioned. The results identified a PV in the BRCA2 gene (*c.2808_2811delACAA*). Her paternal grandfather had lung and prostate cancer at 78 years old. We conducted cascade testing on her mother and brother, both of whom tested negative for PVs. The father declined genetic testing, and no other paternal relatives have been tested. It is possible that the variant was inherited from her father, though it could also be a de novo mutation. The absence of women in her father’s family may explain the lack of breast or ovarian cancer cases.

In contrast, for case 2548 with a detected *BRCA2* PV (*c.5110_5113delAGAA*), we observe a traditional family history indicative of breast/ovarian cancer syndrome. There are three instances of breast and ovarian cancer on her mother’s side, with the cancers manifesting at progressively younger ages in each generation. Her mother was diagnosed with breast cancer at 60 years old, while her first cousin was diagnosed in her 40s. Cascade testing conducted on her mother confirmed the presence of the same genetic variant.

**Figure 3:**
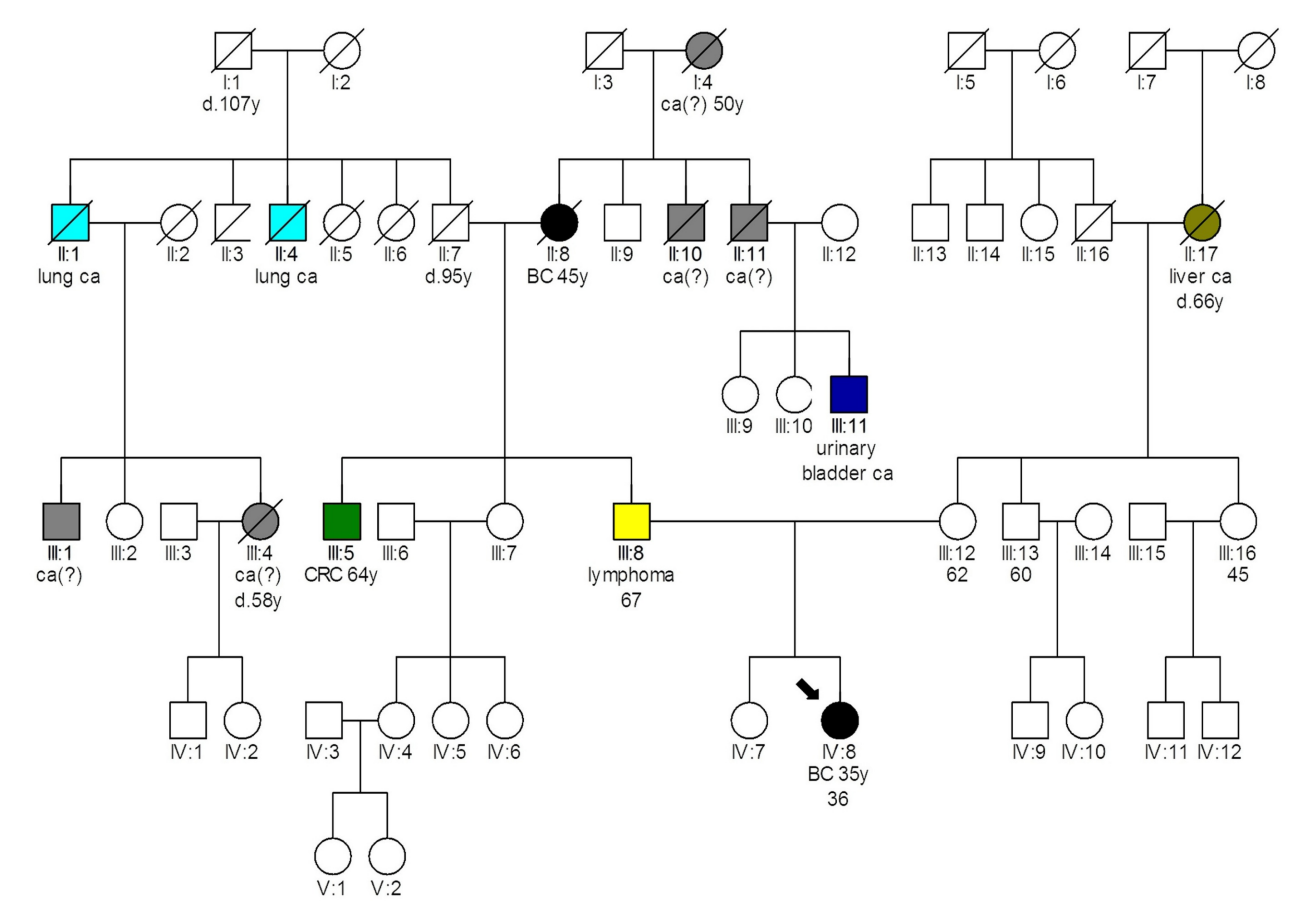
Pedigree of patient 2469 carrying the CHEK2 p.Gly167Arg variant Latin numbering denotes the generation; d: death; y: age in years; Ca: Cancer; Ca(?): Cancer of unknown primary location; BC: Breast cancer; CRC: Colorectal cancer

In the pedigree of patient 2469 (Figure 3), who was diagnosed with breast cancer at 35 and carries the *CHEK2 p.Gly167Arg* a likely PV, we see numerous malignancies on the father’s side. The father had lymphoma at 67, and the father’s brother developed colorectal cancer at 64. The paternal grandmother was diagnosed with breast cancer at 45, and her two brothers, along with their mother, had malignancies of unknown primary sites. Additionally, in the grandfather’s family (father’s side), there are four more cases of cancer, including two lung cancers and two of unknown primary origin. On the mother’s side, there is a single instance of hepatocellular carcinoma in the grandmother. Patient 2469 had IDC, grade III, 5 mm, with no axillary lymph node infiltration (0/3) (stage T1aN0M0), ER/PR positive, HER2 negative, and ki67 as 25% [17]. She received adjuvant radiotherapy and is currently on hormonal therapy, remaining disease-free for eight years. Screening of her parents revealed that she inherited the variant from her mother, contrary to the family history.

**Figure 4:**
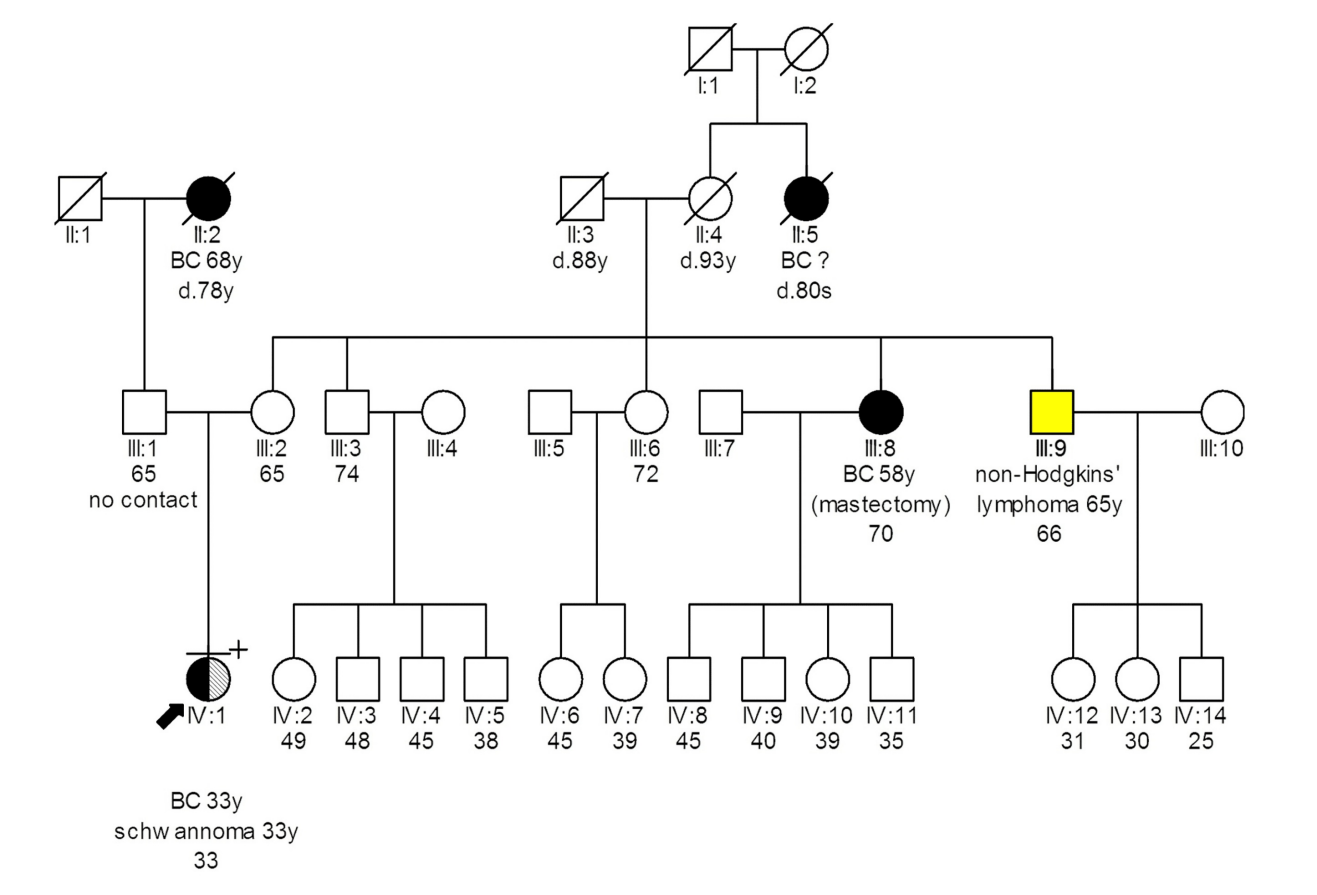
Pedigree of patient 9794181 carrying the TP53 c.473G>A(p.Arg158His) mutation Latin numbering (in example: I, II, III, IV) denotes the generation, d: death, y: years old, Ca: Cancer, ca(?): Cancer of unknown primary location, BC: Breast Cancer, no contact: the patient has no contact with the specific family member

Patient 9794181 was diagnosed with right breast cancer at 33 years old. A PV in *TP53 *(*c.473G>A *(*p.Arg158His*)) was found. She underwent a bilateral mastectomy with right axillary lymph node dissection. Pathology examination reported a multifocal IDC in the right side, grade 3, with greatest dimension 2.5 cm, and 22 positive axillary lymph nodes out of 29 excised (stage T2N3M0) [17]. IHC examination revealed an ER/PR as positive, HER2 as negative disease, with ki67 as 30%. Thereafter she received adjuvant chemotherapy, and she is currently receiving hormonal therapy. One year after diagnosis, she is free of disease. Her mother’s sister (aunt) had breast cancer at 58 years old. Her paternal grandmother was diagnosed with breast cancer at 68 years. In her personal history, she has a spinal neurosurgery with multiple neurinoma. 

## Discussion

We have tested 146 patients for germline variants. We have found 10 PVs in *BRCA1*, 3 PVs in *BRCA2* and 1 VUS, 3 PVs in *CHEK2* and 1 VUS, 1 PV in *TP53* gene, 1 VUS in *MSH2* gene, 1 VUS in *BRIP1* gene (Table 3). Our cumulative variant data showed deleterious variants in *BRCA1* or *BRCA2* in 13 cases (8.9%). We should underline that 70% (7/10) of all variants in *BRCA1* are located at the 3’ end of the gene (exons 20-24) due to founder effects [11].

Detailed guidelines for the genetic assessment and management of high-risk patients are included in the latest version of National Comprehensive Cancer Network (NCCN) clinical practice guidelines in oncology [18].

The *CHEK2* gene serves as a tumor suppressor, is vital for controlling cell cycle checkpoints, initiating DNA repair processes, and promoting apoptosis. Its essential roles in cellular regulation mean that inherited PVs of *CHEK2* are linked to a heightened risk of developing breast cancer [2,19,20]. PVs in this gene confers an absolute risk for breast cancer of 20-40%, but evidence for risk reducing mastectomy is insufficient and management is based on family history [18]. 

*CHEK2* variant *c.1100delC*, found in case 1927, is a founder variant across Europe, while it is less common in southern Europe, including breast cancer patients of Greek ancestry (0.16%) [21,22]. The other *CHEK2* variant *c.499G>A *(*p.Gly167Arg*), found in two cases, is a single nucleotide variant, classified as pathogenic/likely pathogenic in ClinVar [23].

Germline variants in the *TP53* gene are associated with Li-Fraumeni syndrome, which increases the risk of developing various cancers at an unusually young age, such as breast cancer, sarcomas, brain tumors, and adrenocortical carcinomas. For individuals with *TP53* germline PVs, a specific surveillance protocol is recommended. This protocol includes annual breast and brain MRIs, along with full-body MRI without gadolinium enhancement. It also involves clinical examinations with blood pressure monitoring, abdominal ultrasound, and urine steroid measurements every 6-12 months, with close attention to signs of virilization or early puberty in children. Notably, individuals with a PV in *TP53* require special management due to their radiosensitivity. Consequently, it is crucial to implement specific systematic surveillance methods for the early diagnosis of neoplasms [18]. Patient 9794181 had no characteristic phenotype suggesting Li-Fraumeni syndrome. Nevertheless, she benefited from 94 gene panel testing finding a PV in a high penetrant gene, altering her medical management. However, it is essential to recognize that somatic *TP53* variants frequently complicate the results of germline testing, especially in older adults and cancer patients. We performed a double check of the finding with targeted testing of exon5 with Sanger DNA sequencing. 

PVs in genes with moderate penetrance, such as *ATM, CHEK2, BRIP1,* and *PALB2*, double the risk of breast cancer. These genes are included in gene panels along with *BRCA1* and *BRCA2* for comprehensive analysis. Based on NGS technology, multigene panel testing allows for the concurrent sequencing of numerous genes, offering a cost-effective and efficient approach to assessing cancer genetics, guided by clinical phenotype [24]. An individual’s personal and family medical background may include various inherited cancer syndromes. As a result, using a tailored multigene panel test that is directed by specific phenotypes observed in their history can be more efficient and cost-effective. This method improves the chances of detecting a pathogenic or likely pathogenic variant in a gene, which can significantly impact medical management decisions for the individual and their at-risk relatives [18].

Nevertheless, selecting a gene panel that evaluates a broad spectrum of moderate or low penetrance genes could result in the discovery of genetic variants that lack medical significance and do not provide the expected cancer prevention advantages. Furthermore, comprehensive multigene testing raises the probability of identifying VUSs. These VUSs introduce complexities that can complicate clinical decision-making and patient counseling [25]. In our investigation, as well as in a prior study, we observed that a patient referred to as 2122, harbored a VUS in the *BRIP1* gene (*BRCA1* interacting protein C-terminal helicase 1), which is implicated in the DNA repair mechanisms associated with *BRCA1 *[2]. The role of this variant on its molecular function and its potential implications for cancer risk are still not fully understood. Such occurrences are relatively common; data from ClinVar indicate that there are currently 933 reported *BRIP1* variants classified as VUS [26]. VUSs should not influence treatment plans, and patients with these variants should be closely observed for any potential reclassification [18]. 

Currently, many breast cancer patients in Greece are unable to undergo genetic testing due to its high cost. It is worth noting that our study on the current state of medical practice in Greece includes a limited number of patients, primarily due to the high cost of genetic analysis. As a result, our findings should be considered preliminary and require validation in a larger patient cohort. However, as technology advances and testing becomes more affordable and accessible through an increasing number of laboratories, more patients will be able to pursue testing, leading to improved identification of gene carriers.

## Conclusions

In summary, this study underscores the significant role of genetic predisposition in the incidence of breast cancer cases. Timely genetic testing is crucial, can inform treatment choices, and can guide clinical management strategies. The results suggest that although family history plays a significant role in the referral process for genetic testing, it should not be the only factor considered. Notably, among the variant carriers in this study, some had a family history of cancer while others did not. Furthermore, the findings indicate that multigene panel testing may be advantageous for all breast cancer patients. The criteria for offering testing are continually expanding. Employing multigene panel testing, which encompasses more than just *BRCA1/2* mutations, is essential for increasing the likelihood of detecting a germline genetic mutation. However, this approach also increases the chances of identifying VUSs. Individuals with PVs should be provided with tailored post-test counseling. VUSs should not influence clinical management, and patients with these variants should be monitored for potential reclassification.
